# Customizable modification of banding with external stenting for arteriovenous fistula flow reduction

**DOI:** 10.1016/j.jvscit.2022.01.003

**Published:** 2022-01-27

**Authors:** Alexandros Mallios, Antoine Gaudin, Alexandra Hauguel, Romain de Blic, Benoit Boura, William C. Jennings

**Affiliations:** a Department of Vascular Surgery, Groupe Hospitalier Paris Saint Joseph, Paris, France; b Department of Surgery, School of Community Medicine, University of Oklahoma, Tulsa, Okla

**Keywords:** AVF, Banding, Flow reduction, FRAME, High flow

## Abstract

We performed a single-center retrospective study of prospectively collected data for all patients who had flow reduction surgery with FRAME FR between November 2020 and January 2021. Ten patients had arteriovenous fistula flow reduction surgery with this technique. One patient had a distal fistula, whereas nine were within the cubital fossa. In nine patients the device was applied over the postanastomotic arteriovenous fistula outflow vein and in one in the preanastomotic radial artery. Technical success was achieved in all patients with a median flow reduction from 2150 to 825 mL/min. There were no wound or device-specific complications.

The development of excessive high flow (EHF) is a common problem for mid-arm arteriovenous fistulas (AVFs) but may also occur in more distal locations, especially in younger patients. EHF-AVFs are associated with high-output heart failure, pulmonary hypertension, and steal syndrome.^1^^,^^2^ AVF flows over 2000 mL/min clearly carry significant risk for cardiopulmonary remodeling and complications.^3^^,^^4^ Other studies found significant clinical risk associated with access flows of 1500-2000 mL/min.^5^^,^^6^ In addition to the systemic effects, EHF-AVFs are associated with aneurysm formation, cephalic arch stenosis, and increased risk of cannulation site bleeding.

The many methods and configurations of flow reduction testify to the difficulty in achieving proper flow reduction. The most commonly performed technique for flow reduction is AVF banding, frequently applied with focal narrowing using polypropylene suture. However, results are highly variable and widely criticized in terms of efficacy and durability. Importantly, once a restriction is placed, only a one-half-millimeter change in diameter results in exponential flow reduction. Poiseuille’s law specifies a 16-fold change in resistance within a conduit by change in radius of the tube. Increasing resistance and flow reduction may also be augmented by adding length to the flow limited segment.^7^

Inadequate flow restriction offers no benefit to the patient, whereas too great a reduction in flow may lead to post-procedure thrombosis. Access thrombosis or EHF recurrence after banding is not uncommon, and aneurysm formation or erosion of the vein wall has been previously reported.^8^, ^9^, ^10^, ^11^, ^12^

Precision banding has dramatically improved the outcomes by real-time inflow measurements (most often brachial artery), before and after the flow restriction procedures.^13^ Such objective flow measurements evaluate the adequacy of flow reduction in real time, allowing for precise adjustment if needed. FRAME FR takes the advantage of both flow restricting components, allowing precise adjustable narrowing of the conduit diameter and lengthening the flow restricting segment when compared with simple suture restriction by banding.

External stenting has shown promising results in stabilizing vein grafts implanted in coronary bypass grafting. By preventing venous dilatation and development of lumen irregularities, external stents were shown to significantly improve flow pattern and reduce intimal hyperplasia.^14^, ^15^, ^16^ We report our initial experience with a modified banding technique, using an external stent device for flow reduction in EHF-AVFs. Although the principle is similar to banding, this device offers the ability for flow adjustment and potentially more durable results.

## Materials and methods

We retrospectively identified all patients who underwent a flow reduction intervention with FRAME FR between November 2020 and January 2021. All procedures were completed in a not-for-profit teaching hospital outpatient setting, under local or regional anesthesia. Intraoperative ultrasound flow measurements evaluated the adequacy of flow reduction. The patients’ blood pressure was maintained to preoperative levels during the flow reduction procedure.

The primary outcomes of the study were technical success, sustained flow reduction, and AVF patency with freedom from intervention during the study period. Secondary outcomes were freedom from surgical site or device-specific complications.

This study was approved by the institutional review board (Comité de protection de personnes-CPP-Nord Ouest II) and is in accordance with the Declaration of Helsinki. Individual informed consent, covering also the publication of relevant articles, was obtained from all patients.

### Device

**Fig 1:**
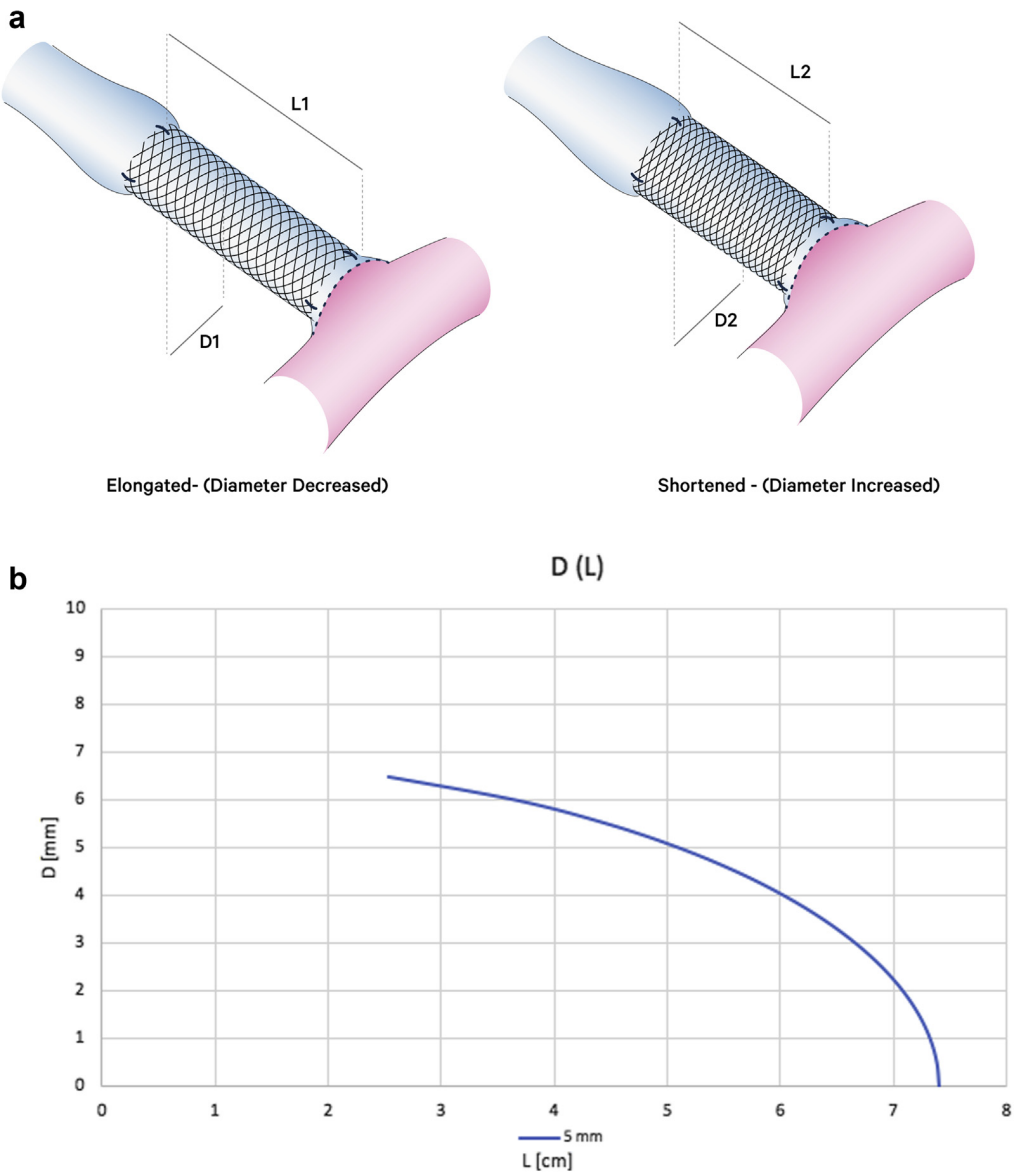

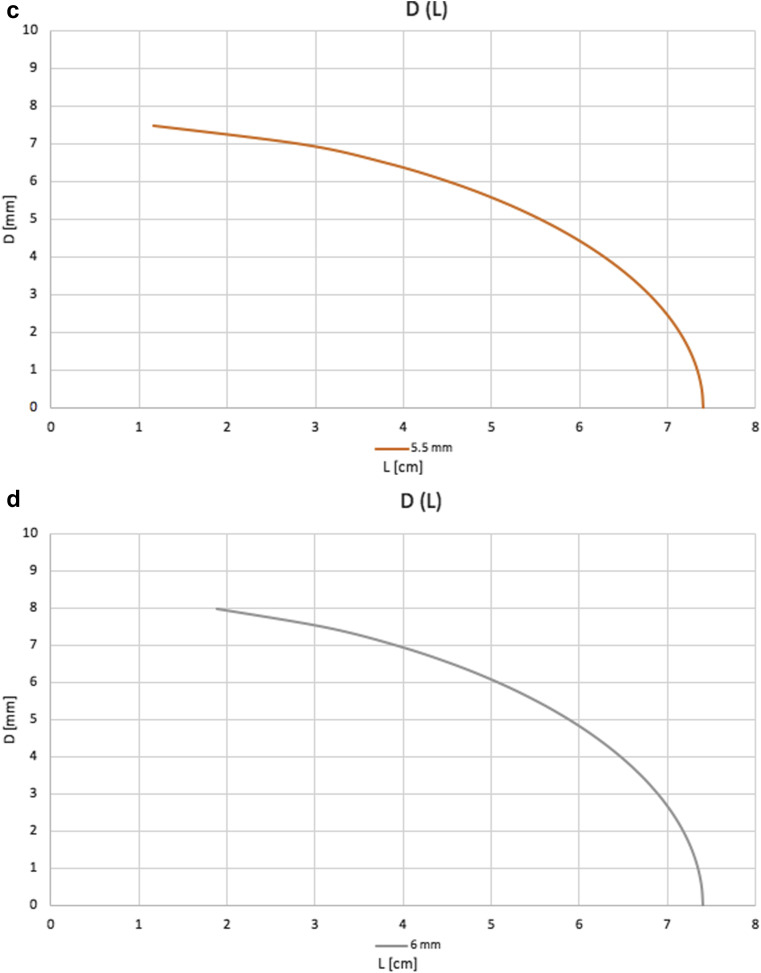
**(a)** Photos of the same device measurements with and without compression. **(b-d)** Length-diameter relationship of 5 cm length type B (blue), C (orange), and D (gray) FRAME FR devices. According to Poiseuille’s law, “R= 8nL/πr^4^,” resistance to flow is directly correlated with length and inversely related to the fourth power of the radius. Therefore, elongation of the device in situ will lead to reduced lumen diameter and a significant increase in flow resistance, whereas shortening the device will lead to an increase in diameter and lower resistance.

FRAME FR has a unique braided structure of cobalt-chromium alloy. It is designed for external reinforcement application over autologous grafts or reconstructed peripheral vessels. The device is available in three different models covering veins with a diameter range of 4.6-8.0 mm and in units of either 10 cm or 20 cm in length. After application, the device can be cut with surgical scissors to fit the desired length. The design of the device is such that when elongated its diameter is reduced and when compressed it is widened (Fig 1). As explained later, this property is used for precision adjustment of the induced resistance for the flow reduction surgery.

### Technique

**Fig 2:**
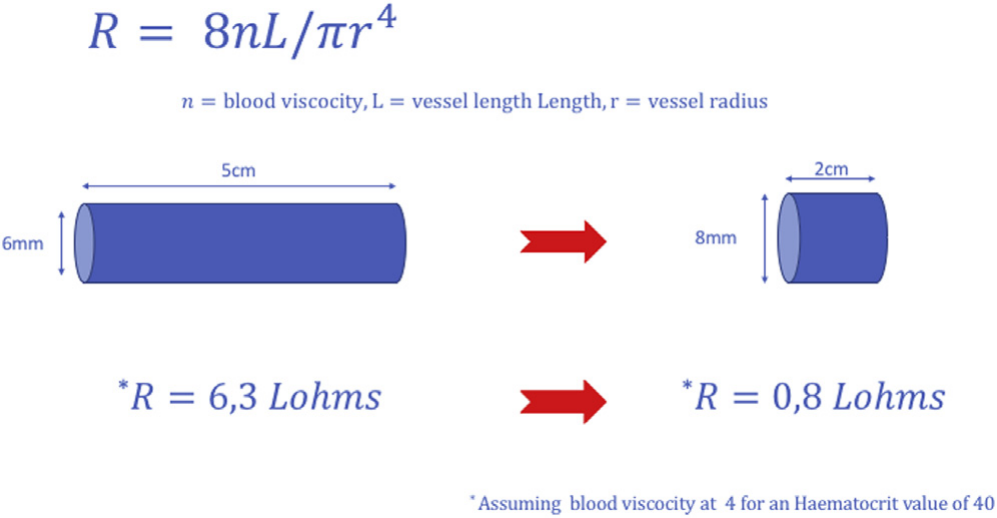
Illustration of the effect of the device on the resistance induced in the circuit. The same device in nominal length of 5 cm (corresponding diameter of 6 mm) inducing a resistance nearly 8 times more important than the same device compressed for a length of 2 cm (diameter of 8 mm). R = Resistance to blood flow.

**Fig 3:**
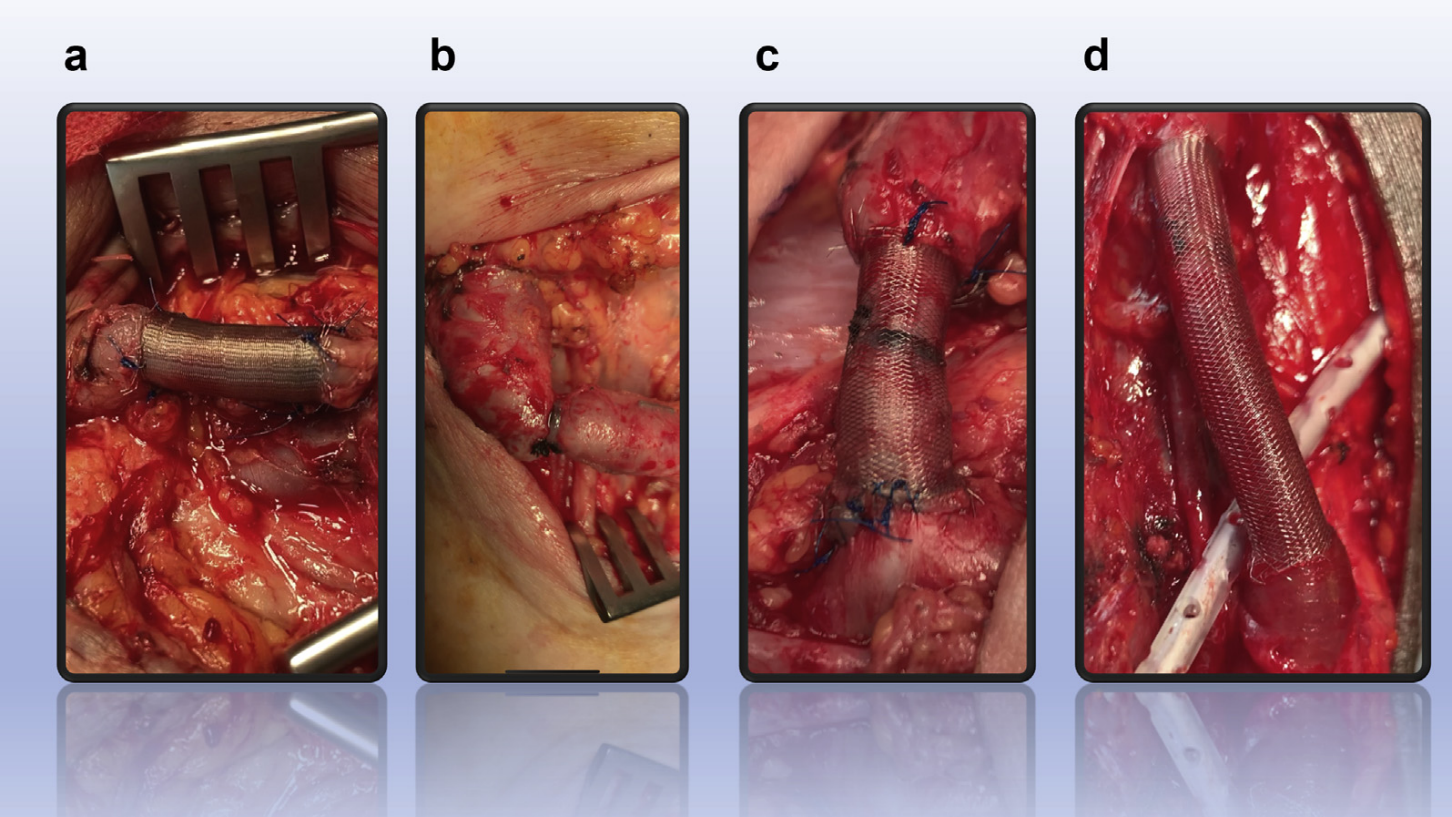
Perioperative images: **(a)** device applied and fixed in a compressed position, **(b)** patient with previous failed banding that came back with high venous pressure, **(c)** the same patient with the Frame applied, **(d)** application of the device on high takeoff radial artery before the anastomosis.

The arteriovenous anastomosis and adjacent outflow segment are dissected free with adequate length of the vessel targeted for the placement of FRAME FR. Most commonly, the device is applied over the first few postanastomotic centimeters of the outflow vein, but placement over an enlarged artery in case of radial or ulnar inflow is also feasible. The vessel is divided at a site that will allow the compressed device to be positioned over one side of the vessel with the aid of nontraumatic forceps. If the vessel diameter is over 1 cm, excessive tissue should be resected longitudinally, and the vessel is recalibrated over a 4- to 5-mm mandrel. FRAME FR should not extend into the cannulation zone. A termino-terminal anastomosis is re-establishing the access with a running vascular suture. Hemostasis is verified with clamp release, and then clamps are replaced to remove pressure and distension. FRAME FR is then extended and positioned over the entire vessel segment and clamps are again released. Ultrasound inflow measurements are repeated, and the thrill is evaluated clinically. We are generally aiming for a barely palpable thrill and a soft feeling fistula at the cannulation segment after clamp removal. Fine adjustments of the length of the device modify the induced resistance (restrictive diameter and length) until the flow is confirmed at the desired level (Fig 2). When this is achieved, the device is secured proximally and distally with interrupted vascular sutures, two proximally and two distally, to maintain the final flow reduction (Fig 3). Without proximal and distal fixation, it is possible that with vessel tension and pulsations the device might shorten and therefore widen, losing the precise adjusted resistance with recurrence of high flow.

## Results

Between November 2020 and January 2021, 10 consecutive patients (7 males) had a flow reduction intervention with FRAME FR in our institution. Nine patients had a history of hypertension and six were diabetic. Nine patients were on dialysis and one had a successful kidney transplant but wished to maintain his fistula. One patient had a distal radiocephalic AVF and the remaining nine individuals had midarm AVFs. Among these, six had brachial artery inflow, two had radial artery inflow with high radial artery takeoff and one had an AVF between the proximal radial artery and the deep communicating vein of the elbow.

**Fig 4:**
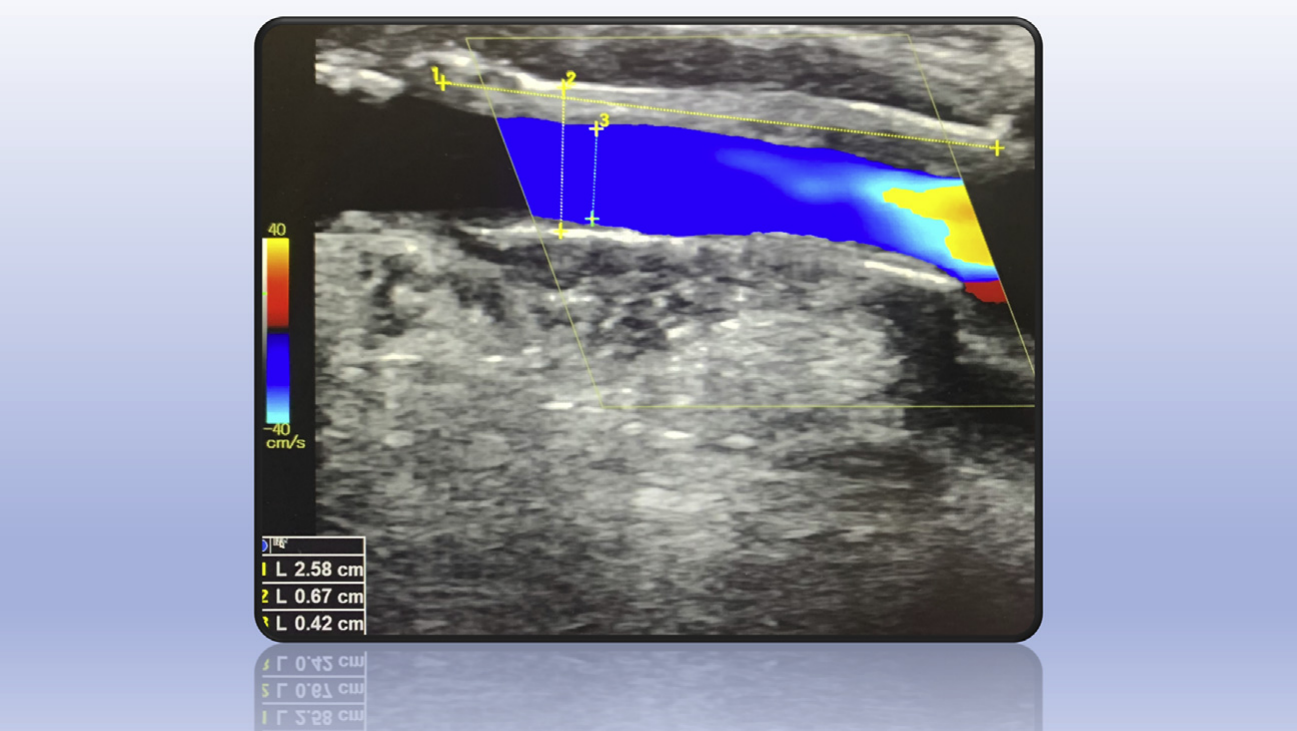
Patient of Fig 3, *a*, postoperatively: note the length of the device, the regular flow within the device, and the external (6.7 mm) and internal (4.2 mm) diameter measurements.

In nine patients, the device was applied over the postanastomotic venous outflow segment and in one over the preanastomotic high radial artery (Fig 3). The median (interquartile range) flow before the procedure was 2150 (1850-2275) mL/min and was reduced to 825 (720-953) mL/min at the latest follow-up visit (mean, 30 days; range, 15-90 days) (Fig 4). In all dialyzed patients, the AVF use was uninterrupted. No patient experienced wound or device-related complications. All patients reported resolution or improvement of symptoms postoperatively, either systemic (reduction of tiredness and shortness of breath), or locally (reduction of venous pressure and duration of bleeding after needle removal). No reinterventions were required during the study period for any patient. Patient characteristics and results are shown in the Table.

**Table:** Patient, arteriovenous fistulas (*AVF*) characteristics, and results

Patient	Age, years	Type of AVF	Artery/Vein^a^	Flow before,^b^ mL/min	Flow after, mL/min
1	59	RC	V	2400	1100
2	83	BC	V	2200	780
3	51	BB	V	2200	970
4	35	RCH	A	2300	900
5	56	RCH	V	1800	1000
6	64	BC	V	2100	700
7	45	BB	V	1700	600
8	63	BC	V	1500	500
9	51	PRA/DCV	V	2000	850
10	49	BC	V	2500	800
Median^c^	53			2150	825

*BB,* Brachiobasilic; *BC,* brachiocephalic; *DCV,* deep communicating vein; *PRA,* proximal radial artery; *RC,* radiocephalic; *RCH,* radiocephalic with high takeoff.

a Referring to the site of application of the FRAME FR.

b Flow measured in the proximal brachial artery.

c Where applicable and in the same units as the respective column.

## Discussion

Dialysis patients with a high-flow vascular access are at significantly greater risk for left ventricular hypertrophy, congestive heart failure, and myocardial ischemia in addition to pulmonary hypertension.^17^, ^18^, ^19^, ^20^, ^21^, ^22^, ^23^ Flow reduction surgery is one of the most frequently performed AVF surgical revisions and is clearly indicated for patients with flow rates greater than 2000 mL/min. Other authors noted significant risk associated with access flows of 1500-2000 mL/min, and these individuals should be considered for flow reduction, particularly in patients with symptomatic heart disease. In addition, EHF-AVFs are often associated with steal syndrome or high venous pressure outflow problems such as cephalic arch and central venous stenosis, aneurysm formation, and prolonged postcannulation bleeding.^24^^,^^25^ In our practice, all patients with flow over 2 L/min regardless of clinical status and patients with flow over 1.5 L/min and signs of poor tolerance (symptoms of heart failure, high-pressure AVF with episodes of bleeding, swelling when a central vein stenosis coexists, etc); have an indication for flow reduction surgery.

Banding is the most common flow reduction strategy. It is well described in the literature and exists in many different variants, but it often fails to demonstrate reproducible clinical success in terms of efficacy and, most importantly, long-term durability.^6^^,^^7^ Although our results and those of others using a precision banding technique are consistent and reliable,^10^^,^^13^^,^^23^^,^^24^ we have encountered failures and complications and acknowledge the space for improvement in technique and durability in flow reduction procedures.^11^^,^^12^ The study by Vaes et al^8^ concluded that banding may not be durable in a substantial percentage of patients. However, their patients who had postoperative access flow <1000 mL/min maintained acceptably reduced access flow. The banding procedures varied in technique, and maintenance of normal blood pressure during flow restriction was not specified. We target post-banding flow to 600-1000 mL/min and find a key element for successful flow reduction to be maintaining the patient’s “normal” blood pressure throughout the procedure, gaining flow measurements that are reflective of the patient’s usual vascular state.

The Poiseuille equations are a guiding principle for the device use and its benefits compared with banding. Unlike conventional banding, which enables us to reduce flow by reducing vessel diameter and increasing resistance, FRAME FR provides the surgeon with more flexibility by controlling both diameter and desired length of the device. According to the Poiseuille equation, flow resistance is highly sensitive to changes in lumen diameter and less sensitive to changes in length of the reconstructed segment. Considering the limitations and potential complication of too aggressive diameter constriction, the ability to determine and adjust in situ the final length of the device provides the surgeon another degree of freedom to fine-tune the final AVF flow that does not exist with conventional banding.

Flow reduction by revision using distal inflow is a more extensive surgical intervention and requires the use of a prosthetic or saphenous graft.^26^ We have recently described an alternative technique using endo-AVF technology and the deep venous system in order to achieve a distalization of inflow.^27^ However, our preference is pre-emptive avoidance of brachial artery inflow and use of the proximal radial artery or the proximal ulnar artery when a distal AVF is not an option.^27^, ^28^, ^29^, ^30^, ^31^

In our experience, steal syndrome rarely occurs with or because of high-flow AVF, most if not all our patients with steal have severe arterial disease, and the AVF aggravates hemodynamic conditions that cannot be tolerated because of poor distal vasculature. In these patients who usually have low-moderate flow AVFs. Proximalization of arterial inflow is our most common intervention.

Shu et al^32^ have shown that by creating arteriovenous fistula using a synthetic graft, the localized high shear stress region at the venous anastomosis correlated with the development of intimal hyperplasia. In another experimental model that simulated compliance mismatch by banding, Okuhn et al^33^ have shown that the difference in radial compliance between the banded and nonbanded segments was not associated with intimal hyperplasia.

An uncommon but recognized issue with focal banding by polypropylene suture is the possibility of suture erosion through the vessel wall over time, losing the flow restriction effect.^12^ Our practice for large-diameter fistulas is to taper the outflow vein and reconstruct the AVF anastomosis to a 3.5-4 mm diameter. We expect the FRAME FR procedure to avoid this issue in the future.

Arguably, a less costly polytetrafluoroethylene band can be used for a postanastomotic banding without the need for section and reanastomosis as performed with FRAME FR. Nonetheless, this technique would only be applicable for moderately dilated AVFs. A severely dilated vessel would still need some form of luminal diameter reduction; otherwise, unpredictable folding and intimal plication may occur and eventually cause fistula thrombosis. Most importantly though, the Polytetrafluoroethylene-mediated banding technique does not offer the possibility of fine-tuning of the induced flow reduction that appears to be unique in our reported technique with FRAME FR utilization.

This early report presents our outcomes and describes the technique, taking advantage of the mechanical properties of the device allowing precise adjustments of the induced resistance (Figs 2 and 3). By adjusting the length and therefore the diameter of the device, we achieve the desired flow reduction, securing the FRAME FR in position when the flow rate objective is met. In our experience, none of the existing flow reduction techniques offers the ability of such reproducible fine-tuning of the resistance introduced into the access circuit. Importantly, the braided cobalt-chromium alloy device is a form of external stenting. It is designed for external stability and we expect the result to be durable, avoiding the need for repeated flow restriction. Of course, our short-term follow-up is not adequate to affirm this hypothesis; however, a prospective study is underway and will address this issue. This study has the limitations of any retrospective review in addition to the limited study population.

## Conclusions

AVF flow reduction with FRAME FR is a novel technique that allows for unique precision adjustments of the induced resistance necessary for reliable treatment of high-flow AVFs. Technical success and safety appear excellent while the metallic structure may allow for long-term flow reduction durability. Prospective studies with longer follow-up are needed to confirm our findings.
